# Maneuvering in the Interval: Reflections on Immanent Entanglements

**DOI:** 10.1007/s10699-020-09766-x

**Published:** 2021-03-31

**Authors:** Heather Wiltse

**Affiliations:** Umeå Institute of Design, Umeå University, Umeå, Sweden

**Keywords:** Philosophy of technology, Things, Relations, Object-oriented ontology, Fluid assemblages, Surveillance capitalism

## Abstract

Both perspective and leverage are needed in order to arrive at a place where it is possible to do the philosophical work required in order to adequately account for our present sociotechnical landscape. One of the key characteristics of this landscape is the collapse of scale, as things become more like *fluid assemblages* and the economic incentives of surveillance capitalism turn ordinary things into surveillance devices tuned for others’ profit. In this context we need a language not only of imagination and humility in the face of countless gaps between things, but also one of entanglement, care, and response-ability.

## Introduction

If one happens to be trapped in a forest, walking around in circles without making any progress, the wise inclination is to try to find a vantage point that is high enough to enable a wider perspective. Such a view can provide external reference points that allow for better understanding one’s own place and navigating in the desired direction. With any luck, one will also find that the insurmountable obstacle between one’s current position and the goal of the quest is actually an illusion, a projection that shifts and shrinks when the light is repositioned. This is roughly the shape of Van Den Eede’s trajectory in trying to mediate between postphenomenology and its critics: ‘empirical’ and ‘transcendental’, ‘small’ and ‘big’ picture views.

It is in a way fitting that he enters the debate in what might be described as a postphenomenological spirit: looking at the relation that is constituted by two entities, and what that relation allows for doing and perceiving. He also plays the role of mediator in a peacemaking sense, looking for a middle ground where (friendly) combatants might be able to lay down arms and find a common way forward. He finds this middle ground in the realm of object-oriented ontology (OOO), which pulls the rug out from under the entrenched positions and reveals that they have been standing on common ground all along while making shadow monsters out of the things that divide them.

Of course, this rather playful description is of an endeavour that is in fact quite serious. At stake is not only finding a broader *perspective*, a better place to stand, but one that can also allow for achieving the necessary *leverage* for doing the philosophical work that our present sociotechnical landscape requires. The central task of philosophy of technology, according to Van Den Eede, is mediating between instrumentalism and determinism, accounting for agency in pragmatic interactions and larger power-laden structures without being pulled entirely toward either. This requires thinking ‘small things’ and ‘big things’ together and seeing their interrelations in ecologies. (Van Den Eede’s ecological sensibilities come out more strongly in his other work, notably in his recent book [2019] interpreting the work of Gregory Bateson in relation to philosophy of technology).

We need to be prepared to provide much-needed critical perspectives on emerging algorithmic, nano-, and other technologies that stretch our sense-making capacities in more ways than one, even as they also enable these capacities. As alluded to in the introduction of Van Den Eede’s paper, we need both perspective and leverage, understanding and action. And we need to find these in the spaces, or the “interval,” between ‘small’ and ‘big’ things.

## Assessing Current Equipment

Let us briefly, then, assess how well we are equipped for this task. One of the strengths of postphenomenology and other empirical perspectives is their practical utility. This can be seen, for example, in the many extensions and variations of Ihde’s ‘I-technology-world’ schemas, and in its practical application (e.g., Hauser et al. 2018). More transcendental perspectives enable sensibilities for Big Things (and Technology), and perhaps (minimal) possibilities to configure an intentional stance in relation to them. However, as Van Den Eede states, it is exactly the intertwinement of these perspectives that calls for study. Through OOO, it becomes possible to gain some humility and step back from an anthropocentric perspective in recognizing that a presence and withdrawal dynamic characterizes all relations, and not only the ones that humans have. Philosophies of access thus go out the window, as they expand to include all things. Perhaps this is the way to get at the intertwinement?

At least the g/Gap is neatly managed. Now what? How to pursue the task of philosophy of technology, looking out of objects into others? What does this vantage point allow us to see, and what does it miss?

One clue to what OOO misses can be found in its natural enemy, materialism. In contrast to the rollicking braggadocio and relative detachment (‘there are gaps everywhere’) of Harman et al., in (new/feminist) materialist (partial) perspectives we find a much different feeling and sensibility: one of entanglement and care, response-ability, becoming-with, and positionality and power. As a countermeasure to the ‘fresh breeze of OOO’ that Van Den Eede lets in, we might bring in the stench of Haraway’s compost pile that she uses to think through the art of living and dying well together in what she terms the Chthulucene (Haraway 2016). Going back to OOO, we could even bring in Morton’s (2013) sense of the oppressive nearness of hyperobjects, stuck to us everywhere. Everything is entangled, and at stake. (This has perhaps never been more clear than it is at the time of this writing in the midst of the coronavirus pandemic in the spring of 2020.)

As Haraway states in her frequently-quoted and memorable phrasing: “It matters which stories tell stories, which concepts think concepts. Mathematically, visually, and narratively, it matters which figures figure figures, which systems systematize systems” (Haraway 2016, 101). Yes, there is always a gap, a withdrawn substance to all things that we cannot access. Yet there is also always connection. We are always entangled, unspeakably remote and oppressively close at the same time in relation to other ‘objects’ in our intertwined processes of becoming in positions of sometimes radically unequal power. Here is the intertwining of micro and macro, structure and agency, presence and (impossibility of) withdrawal.

## Technological Withdrawal, Take Two

But where do we find technology in this newly steamy picture? Here with this question we arrive back at the original problem: do we find technology in prosaic interactions, or in large systems and conditions of possibility? Or, since we recognize that we need to look in that messy middle where both are intertwined, what can we actually grab onto to achieve some leverage?

This brings us to another problem, and another postphenomenological allergy that Van Den Eede identifies: the ontological. Most of the time in either ‘small’ or ‘big’ analysis, the moving parts are more or less known (or assumed) in advance. At the small end, there is a technological tool (hammer, telescope, cell phone…); at the big end, social structures, power, language, ideology, and the like. One major current problem is that many such existing categories no longer work—as in, they no longer allow for achieving the necessary leverage to do significant work.

Connected algorithmic technologies and planetary-scale computation (Bratton 2015) are in many ways fundamentally different from our previous technologies and associated sociotechnical landscapes. Importantly for present purposes, connected things are both discrete objects we can interact with and connected systems and mediating (infra) structures (Wiltse 2017) at the same time. As my colleague Johan Redström and I have argued, they are more like *fluid assemblages* than more traditional and stable physical objects; and they require making new theory that can more adequately account for their character and scaffold incisive analysis and thoughtful action (Wiltse et al. 2015; Redström and Wiltse 2019a, b).

Things that are fluid assemblages entail a significant collapse of scale: things we can hold in our hands also contain, and are part of, larger assemblages. And in order to better understand what this means practically in our current sociotechnical context, we can turn to the work of Shoshana Zuboff (2019). She identifies the ‘laws of motion’ behind current sociotechnical forms, which also links up concrete interaction and conditions of possibility and ongoing operation. Her diagnosis and extensive analysis of *surveillance capitalism* as the current mode of capital accumulation reveals that connected devices now provide the function of serving as supply routes for behavioral data of ever-expanding scope, scale, and granularity. This behavioral data is used to create prediction products that are sold in behavioral futures markets to (corporate, government) actors willing to pay for specific outcomes. The surest form of prediction being control, we also see that many things and systems are designed to nudge and herd us toward intended behavior. The perhaps most striking example so far is Pokémon Go, the augmented reality game that Zuboff shows to be a proof of concept aimed at driving behavior in the physical world (in this case, herding people to particular locations) through digital interactions.

In this current context where many if not most companies are following the lead of Google and Facebook in pursuing surveillance revenues based on behavioral data from personal devices, ‘smart cities’, and so on, it becomes necessary to revisit existing frameworks, orientations, and even cherished dichotomies that order conceptual approaches and terms of debate. On the practical side, one such framework that does not fare well, at least on its own, under current conditions is that of (user) experience. Much of what (connected) things are and do is, by design, not present to experience during use (Wiltse 2020b; Redström and Wiltse 2019a). Thus, a framework in which experience is the main focal point misses huge and crucial pieces of the picture—and this is a picture in which larger issues of power, knowledge, and political economy are central to the very practical workings and consequences of larger systems. And it is not only small things coming together to form big things, but also ‘big things’ and systems making and sensing through ‘small things’ we can interact with. The micro and macro are intertwined.

## Gaps and Immanent Entanglements in Assemblages

How then to follow this entwinement? The (sensible) object empathy that Van Den Eede prescribes can be one part of a viable approach; although imaginatively following a visual recognition bot (his example) will arguably not lead to the most incisive commentary possible on the overall situation. But another object-oriented approach that is explicitly oriented toward politics and power is that of Levi Bryant (Bryant 2014). He develops *onto-cartography* as a method of concretely investigating “structural couplings between machines [his broad term for objects] and how they modify the becomings, activities, movements, and ways in which the coupled machines relate to the world about them. It is a mapping (cartography) of these couplings between machines (onta) and their vectors of becoming, movement, and activity” (Bryant 2014, 35). This he develops into a “geophilosophy” that aims “to provide us with the means to constructively intervene in worlds so as to produce better ecologies or assemblages” (257). Although Bryant does not really engage with philosophy of technology, geophilosophy might be a good aim and practice for this field as well.

How to proceed, then, from within assemblages, with gaps and entanglement everywhere; and also gaps between existing categories and current realities, and between how connected things present themselves and what they actually do under surveillance capitalism? I suggest that one possible approach has to do with maneuvers in the interval that Van Den Eede identifies, looking out from one situated place onto another.

One set of necessary maneuvers has to do with analysis and sensemaking—engaging in order to see something, from a particular perspective. And, indeed, engagement is always necessary when dealing with fluid assemblages due to their *multi-instability* and *multi-intentionality*, as they change over time in relation to different contexts, users, and user positions (Redström and Wiltse 2019b; Wiltse 2020). *Relation* is also a key concept for sorting out the character and consequences of contemporary connected things, and trying to figure out what they mean to and for us (Wiltse 2020b). We might also think of maneuvers in the more militaristic sense as large-scale exercises in service of particular goals, as in how powerful actors exercise and consolidate their power in and through everyday technologies (and there is the micro and macro again). More importantly, as both scholars and users of ordinary technologies in the technosphere (Puech 2016), we must develop skilful tactics through small acts of agency while maneuvering in larger environments, seeking ways to tune the things and systems that would tune us.

Van Den Eede concludes his piece by asserting that it is “better to speak of countless ‘before’s’” than of a Gap before essence. The ‘gap’ is smeared out to apply to all things. But we also need to account for new kinds of things (ones that have not yet shown up in even the most imaginative Latourian litany). When existing categories break down and existing orientations and debates lose relevance, it becomes necessary to develop new conceptual tools that are more adequate to the task at hand (Redström and Wiltse 2019b). To an appropriate awareness of the gaps between all things, and corresponding humility and curiosity, we might also add a sensibility for immanent entanglements. We need a language of not only gaps and detachment, but also of connection, care, and response-ability. Yes, there are countless ‘before’s’, but also *nows*—nows that require questioning about the current and possible future roles of technology in posthuman good lives in the Anthropocene/Capitalocene/Chthulucene. In which histories and futures do we find ourselves, *now*?
